# Multidrug resistance and virulence profile of the commensal *Proteus mirabilis* isolated from a native Iraqi frozen chicken carcass

**DOI:** 10.1016/j.jgeb.2025.100490

**Published:** 2025-04-26

**Authors:** Zaid A. Thabit, Zahraa A. AlShaheeb, May Ridha Jaafar, Safaa A.S. Al-Qaysi, Sana M.H. Al-Shimmary

**Affiliations:** a Al-Nahrain University, Biotechnology Research Center, Baghdad, Iraq; b Iraqi Ministry of Health, Baghdad, Iraq; c Department of Forensic Biology, Higher Institute of Forensic Sciences, Al-Nahrain University, Baghdad, Iraq; d Biology Department, College of Science for Women/University of Baghdad, Baghdad, Iraq

**Keywords:** *Proteus mirabilis*, Resistance genes, Virulence genes, Food poisoning, Phylogenetic tree

## Abstract

This study aimed to determine the prevalence of *Proteus mirabilis* in frozen chicken carcass from local slaughterhouse. It assesses the activities of nine antimicrobial agents and the presence of antimicrobial resistance genes and virulence genes. Thirty samples were collected from five local Iraqi companies. and then the antibiotic-resistance genes and virulence factor-related genes were detected via polymerase chain reaction (PCR). The results revealed that Nine *P. mirabilis* isolates were recovered, and the majority of the isolates were resistant to both nalidixic acid and azithromycin at a ratio of (100 %), followed by trimethoprim-sulfamethoxazole (*sul1*) (88.8 %), whereas the isolates were susceptible to imipenem and meropenem, and both ceftazidime and cefotaxime were efficient at a ratio of (88.8 %). All the isolates (100 %) were resistant to at least three classes of antibiotics and were classified as multidrug resistant. The PCR results indicated that the most common resistance genes were DNA Gyrase Subunit A Gene (*gyrA*) (100 %), Dihydropteroate Synthase Gene (*sul1*) (88.8 %), and Florenicol Resistance Gene (*floR*) (88.8 %), followed by Aminoglycoside N-Acetyltransferase Gene (*acc (6′)-lb*) (44.4 %) and Macrolide Phosphotransferase Gene (*mphA*) (33.3 %). In addition, the virulence genes Zinc Metalloprotease A Gene (*zapA*), Uridine Monophosphate Synthase Gene (*uraC*), Histone-Modifying Protein A Gene (*hpmA*), Flagellin A Gene (*flaA*), Anti-Sigma Factor *RsbA* Gene (*rsbA*), and Multidrug Resistance Protein A Gene (*mrpA*) were found in the same proportion (100 %) of all *P. mirabilis* isolates. Our study emphasized that *Proteus mirabilis* has a high frequency of antibiotic resistance as a multidrug resistance pattern and furthermore demonstrated a high level of virulence factor gene detection, which might be a threat to food safety and human health. The phylogenetic tree analysis of the *P. mirabilis* isolates from chicken meat revealed high similarity to the database strain.

## 1 Introduction

Food poisoning continues to be a major source of concern for the public health sector. The problem occurs when someone consumes contaminated food such as meat, vegetables, and seafood ([67], [68]); moreover, kitchen surfaces and workers’ hands may contaminate each other in a cyclical pattern because cross-contamination should not be ignored since it can cause serious infection.[58], [5] In the last two years in Iraq, poultry meat production has increased without food quality assurance from the farm slaughterhouse production stage until it reaches the consumer. In reality, bacteria present in the avian microbiome and slaughterhouse, particularly in equipment used to handle carcasses, cuts, and byproducts, can infect meat during and after chicken slaughter.^47^ *Proteus mirabilis* is a causative agent of food poisoning in many cases around the world.^23^ *Proteus mirabilis* is a gram-negative bacillus that belongs to the *Enterobacteriaceae* family and is widely spread in the environment and the gastrointestinal tract of humans and animals.[14], [10] In the last several decades, many cases of food poisoning have occurred because of the consumption of food contaminated with *P. mirabilis*.[14], [23] In August 2008, in China, particularly in Beijing, a food poisoning incident involving thirteen individuals, the contaminated food was stewed pork balls in brown sauce, and similar cases have been reported in the United Kingdom, Bristol Royal Infirmary, with the consumption of tomato puree. Abdominal pain, diarrhea, nausea, and dizziness are the most common symptoms in patients with food poisoning.[23], [63] The presence of *Proteus* spp. in chicken feces with other enteric bacteria, including *Escherichia coli*, indicates its role as a constituent of the normal intestinal microbiota.^18^ This facilitates the dissemination of these bacteria to slaughter lines and cross-contamination, especially during the carcass evisceration phase. Certain bacterial contaminants may endure storage after carcass processing. As a result, consumers must be informed that poultry meat might be a source of microbe transmission. Therefore, the disinfection of handling instruments following the processing of meat is crucial to prevent any cross-contamination.^20^

Antimicrobial resistance (AMR) has become an increasing and significant threat to global health.[65], [57] A 2016 report projected that global deaths resulting from infectious diseases attributed to AMR would increase from 0.7 million to 10 million by 2050, accompanied by a projected inaction cost of approximately US$100 trillion between 2016 and 2050.^54^ *P. mirabilis* has been recovered from many food items, especially those of animal origin, such as chicken flesh, with certain isolates exhibiting multidrug resistance (MDR).[27], [64]

Polymerase chain reaction (PCR) is an exceptionally sensitive technique utilized for the detection of specific pathogens in clinical samples. A variety of PCR assays have been developed to identify and detect pathogens affecting ducks, as well as zoonotic bacterial pathogens.^19^ *P. mirabilis* pathogenicity in humans may be associated with several virulence factors. *P. mirabilis* bosses virulence factors, including flagella, urease, pili, hemolysin and metalloproteinases, which increase the pathogenicity of *P. mirabilis* through different mechanisms and can help it colonize and destroy tissues and escape immunity[12], [29]. One of the most noticeable characteristics of *Proteus* spp. is their capacity to swarm on solid surfaces. Numerous genes are involved in the swarming phenomenon, and the anti-sigma factor *RsbA* gene (*rsbA*) is essential for swarming regulation^44^. *P. mirabilis* fimbrial protein uridine monophosphate synthase gene (*uraC*), which adheres to desquamated uroepithelial cells, was shown to be organized as pili on the surface of bacteria^66^. The *zap* operon, which is expressed by the genes zinc metalloprotease A gene (*zapA*), *zapB*, *zapC*, and *zapD*, is critical for the synthesis of proteases, particularly *zapA*, which regulates IgA protease expression during swimmer cell differentiation to swarmer cells^62^. *P. mirabilis* has been shown to exhibit a wide range of fimbriae. The important type is MR/P fimbria, which is encoded by the genes Multidrug Resistance Protein A Gene (*mrpA*), *mrpB*, *mrpC*, *mrpD*, *mrpE*, *mrpF*, *mrpG*, and *mrpI*. The *mrpA* gene adds multiple virulence elements to pathogenicity, including bacterial adhesion to epithelial tissue, biofilm formation, and swarming phenomena^45^. Another crucial factor is the production of the hemolysin histone-modifying protein A gene (*hpmA*) by *P. mirabilis*, which bacteria use to damage kidney tissues^16^, and the flagellin A gene (*flaA*), which is essential for flagellar biosynthesis and motility^17^.

*P. mirabilis* has been linked to foodborne illness and may pose a public health risk to society because of its strong relationship with several human infectious illnesses^24^. The objectives of this study were to evaluate the presence of *P. mirabilis* among frozen Iraqi chickens and their susceptibility to antibiotic drugs and to perform PCR detection of the florenicol resistance gene (*floR*), aminoglycoside N-acetyltransferase gene (*acc(6′)-Ib*), macrolide phosphotransferase gene (*mphA*), DNA gyrase subunit A gene (*gyrA*), dihydropteroate synthase gene (*sul1*) and virulence genes *zapA*, *uraC*, *hpmA*, *rsbA*, *flaA*, and *mrpA*.

## 2 Materials and methods

### 2.1 Sample collection

From October 2020 to May 2021, thirty samples of frozen chicken meat were collected from five Iraqi poultry production companies and divided into three groups: chicken breasts, chicken thighs, and whole nine-piece chickens (ten items from each group). The collection of samples was performed in Baghdad.

### 2.2 Bacterial isolation and identification

*Proteus mirabilis* bacteria were recovered from poultry flesh in accordance with ISO 6579-1:2017(E). This protocol is for the isolation of *Salmonella* spp. bacteria^36^, but we found that it could be used to isolate several types of *Enterobacteeriacae* spp., including *P. mirabilis,* when *Salmonella was isolated* in another published study^11^. The ISO protocol was as follows: Twenty-five grams of each final chopped sample was inoculated into a sealed flask containing 225 ml of sterile buffered peptone water (Himedia, India) and then incubated at 37 °C for 18 h. Subsequently, 0.1 ml of the preenrichment media was inoculated into 10 ml of Rappaport Vassiliadis broth media (Himedia, India) and incubated at 41 ± 0.5 °C for 24 h with thorough mixing to enhance the culture. Then, a loopful of enriched suspension was inoculated onto two types of selective media (xylose lysine deoxycholate and *Salmonella-Shigella* agar), and the agar plates were incubated at 37 °C for 24 h. Suspected isolates such as *Proteus* were biochemically identified by using the VITEK-2 system (bioMérieux, France).

For black colonies with swarming phenomena, genomic DNA was extracted according to the methods of the HiPer® Bacterial Genomic DNA Extraction Teaching Kit (solution-based), India. The purified DNA was transferred to 1.5 ml microcentrifuge tubes and stored at −20 °C until use. A conformation test was performed by amplifying the *16S rRNA* gene via PCR with the primer pairs shown in Table 1 (Biometra, Germany) through the use of 2X master mix for the PCR process (Promega GoTaq® G2 Green Master Mix, USA).

**Table 1:** Oligonucleotides used in this study.

**Target gene**	**Gene type**	**Primer sequence (5′- 3′)**	**Amplicon size**	**Annealing temp (C°)**	**Reference**
*16S rRNA*	Confirmatory	F primer: GTCAGATGTGAAAGCCCCGA R primer: TCTTTTGCAACCCACTCCCA	838 bp	56 °C	This study
*floR*	Antibiotic resistance	F primer: GCCTTTGTTGCGTTTCGTCT R primer: CGCGAAGGCCAAGCTAAATC	456 bp	58 °C	This study
*acc (6′)-lb*	Antibiotic resistance	F primer: GGACGGAAGGTGGGAAGAAG R primer: GACGGGTCCGTTTGGATCTT	165 bp	58 °C	This study
*mphA*	Antibiotic resistance	F primer: TCGTTGCCTATCCCATGCTC R primer: GTAGAGATCGCCATGCACCA	335 bp	58 °C	This study
*gyrA*	Antibiotic resistance	F primer: ACTGAAGCCAGTACACCG R primer: CAAATCCGCCAGCAGTTC	295 bp	54 °C	This study
*sul1*	Antibiotic resistance	F primer: ATGGTGACGGTGTTCGGCATTCTGA R primer: CTAGGCATGATCTAACCCTCGGTCT	840 bp	65 °C	This study
*zapA*	Virulence gene	F primer: ACCGCAGGAAAACATATAGCCC R primer: GCGACTATCTTCCGCATAATCA	540 bp	58 °C	^60^
*uraC*	Virulence gene	F primer: GTTATTCGTGATGGTATGGG R primer: GTAAAGGTGGTTACGCCAGA	317 bp	56 °C	^60^
*hpmA*	Virulence gene	F primer: TGGTATCGATGTTGGCGTTA R primer: GTGGTGCCCACTTTCAGATT	717 bp	56 °C	^9^
*flaA*	Virulence gene	F primer: AGGATAAATGGCCACATTG R primer: CGGCATTGTTAATCGCTTTT	417 bp	52 °C	^9^
*rsbA*	Virulence gene	F primer: TTGAAGGACGCGATCAGACC R primer: ACTCTGCTGTCCTGTGGGTA	467 bp	58 °C	^2^
*mrpA*	Virulence gene	F primer: GAGCCATTCAATTAGGAATAATCCA R primer: AGCTCTGTACTTCCTTGTACAGA	648 bp	58 °C	^5^

### 2.3 Antimicrobial sensitivity testing

The antimicrobial susceptibility of the identified isolates was then tested via the Kirby–Bauer disk diffusion technique on Mueller–Hinton agar by following the Clinical and Laboratory Standards Institute's guidelines for nine drugs from various families as previously outlined^1^. Phenolic group: chloramphenicol (30 µg); aminoglycoside: gentamicin (10 µg); quinolone: nalidixic acid (30 µg); carbapenems: meropenem (10 µg) and imipenem (10 µg); sulfonamides: trimethoprim-sulfamethoxazole (30 µg); macrolides: azithromycin (15 µg); cephalosporins: ceftazidime (30 µg) and cefotaxime (30 µg). *Escherichia coli* (ATCC 25922) was utilized for quality control purposes. The inhibition zone was measured after 24 h of incubation, and the isolates were interpreted as susceptible, intermediate, or resistant according to the CLSI^1^ standards.

### 2.4 Detection of antibiotic resistance genes and virulence-related genes in *P. mirabilis* isolates

The PCR amplification of virulence and antimicrobial resistance genes was performed via specific primers, and the annealing temperatures for each gene are listed in Table 1. The PCR Master Mix (Bioneer’s AccuPower PCR PreMix, Korea) was used. The conditions of PCR amplification were as follows: initial denaturation for 5 min at 95 °C for 5 min; 30 cycles of denaturation at 95 °C for 30 s; annealing temperatures are shown in Table 1 for 40 s; and extension at 72 °C for 30 s. The final extension was performed at 72 °C for 10 min.

### 2.5 Gel electrophoresis

Agarose powder (1 % w/v) was dissolved in 1X TBE buffer. The mixture was microwaved and allowed to cool at 50 °C before 8 µl of Red-safe dye (iNtRON/Korea) was added to the agarose solution and poured into the tray. The gel was left to solidify, and the comb was removed. The PCR products were electrophoresed to allow standardization via a DNA ladder (Bioneer, Korea) from 1 kb to 100 bp as a molecular marker.

### 2.6 Sequence analysis

For DNA sequencing, the Sanger sequencing method was used. *16S rRNA* gene amplification was carried out via conventional PCR via GoTaq® G2 Green Master Mix (Promega, USA). The PCR conditions and primers used in this study are listed in Table 1 and Table 2. Furthermore, the amplicon of the identified *16S rRNA* gene (45 µl) with forward and reverse primers (838 bp) was sent to Macrogene Comp. (Korea). The forward and reverse DNA sequences for each sample were then aligned via BioEdit and Mega7 software to create consensus sequences. In addition, each analyzed sequence isolate was compared with the NCBI gene bank database to assess similarity matching via the BLAST website tool.

**Table 2:** Prevalence of *P. mirabilis* in collected food samples.

Processing points	Number of sample	Positive result of isolate
Chicken whole 9 pieces	n = 10	3 (30 %)
Frozen chicken breast	n = 10	3 (30 %)
Frozen chicken thighs	n = 10	3 (30 %)

### 2.7 Phylogenetic tree

This research employed Molecular Evolutionary Genetics Analysis (MEGA) software, version 11, to examine the evolutionary relationships among *P. mirabilis* strain sequences and construct a phylogenetic tree. We utilized PCR to obtain *16S rRNA* from three novel bacteria in both orientations during this study. MEGA software was used to align all the sequencing data from the *16S rRNA* gene via the forward and reverse complements of the reverse primer. The gene sequences were aligned separately. The supplementary samples used for phylogenetic analysis were sourced from GenBank. We subsequently analyzed the sequences obtained from our research dataset with the reference sequences supplied from GenBank. The history of evolution was inferred via the neighbour-joining method.^60^

## 3 Results

### 3.1 Bacterial isolation and identification

The prevalence rates of *P. mirabilis* in food samples are listed in Table 2. Among the 30 chicken meat samples from the three processing points (chicken whole 9 pieces, frozen chicken breast, and frozen chicken thighs), nine (30 %; 9/30) isolates were positive for *P. mirabilis*. A biochemical test revealed a high identity percentage (94–99 %). All the *P. mirabilis* isolates were subjected to molecular identification via *16S rRNA* detection, and the results revealed that the genes detected were present in all the *P. mirabilis* isolates (100 %; 9/9).

### 3.2 Antimicrobial susceptibility test

The effects of nine different antimicrobial agents against our *P. mirabilis* isolates were investigated*.* In this study, all the isolates (100 %) were multidrug resistant (MDR), as shown in Table 3.

**Table 3:** Antibiotic resistance patterns of P. mirabilis susceptible (S) or resistant (R) to nine antibiotics chosen according to the CLSI^1^.

**Sample no.**	**Species**	**NA**	**STX**	**CHL**	**GEN**	**MEM**	**CEFZ**	**CTX**	**AZM**	**IMP**	
1	*P. mirabilis*	R	R	S	R	S	S	S	R	S	MDR
2		R	S	R	S	S	S	S	R	S	MDR
3		R	R	R	R	S	S	S	R	S	MDR
4		R	R	R	R	S	S	S	R	S	MDR
5		R	R	R	R	S	S	S	R	S	MDR
6		R	R	R	R	S	S	S	R	S	MDR
7		R	R	R	R	S	S	S	R	S	MDR
8		R	R	R	R	S	R	R	R	S	MDR
9		R	R	R	R	S	S	S	R	S	MDR

**Fig. 1:**
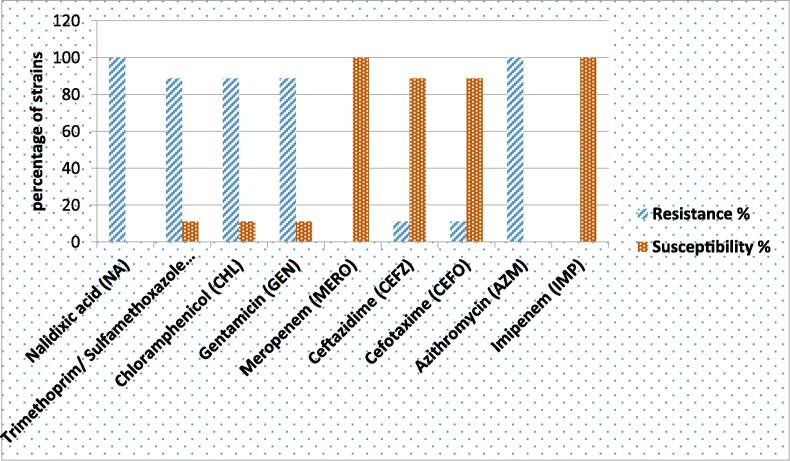
Antibiotic effect patterns among *P. mirabilis* isolates that are susceptible or resistant to antibiotics.

The sensitivity patterns of the *P. mirabilis* isolates revealed that all the isolates were susceptible to meropenem and imipenem (100 %; 9/9), followed by ceftazidime and cefotaxime (88.8 %; 8/9). Both nalidixic acid and azithromycin (100 %; 9/9) were used, followed by trimethoprim/sulfamethoxazole and chloramphenicol (88.8 %; 8/9), as shown in Fig. 1.

### 3.3 Antibiotic resistance and virulence gene detection

**Fig. 2:**
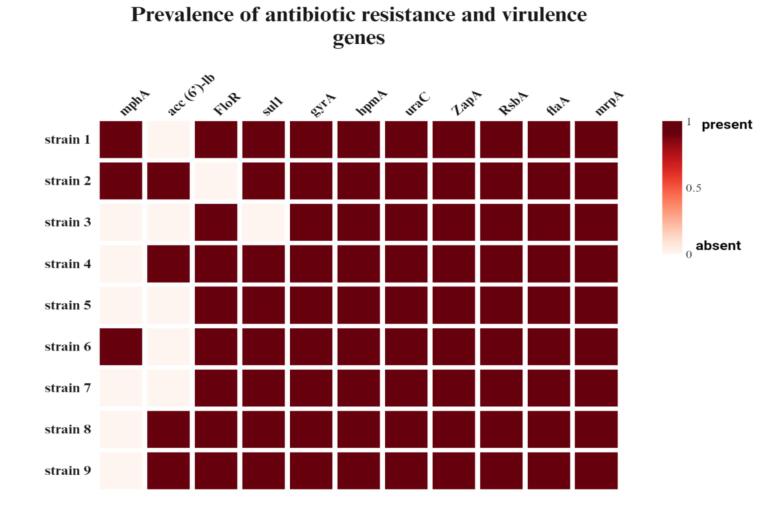
Prevalence rates of antibiotic resistance genes and virulence genes in *P. mirabilis* isolates. Created in https://BioRender.com.

To detect the presence of antibiotic resistance genes for most antibiograms showing resistance among *P. mirabilis* isolates, the PCR results revealed that the isolates produced PCR amplicons for all primers with the same typical target sizes, and the prevalence rates of *floR, acc (6′)-lb, mphA, gyrA, and sul1* in *P. mirabilis* are listed in Fig. 2. The virulence genes *zapA, uraC*, *hpmA, flaA, RsbA* and *mrpA* were detected in all *P. mirabilis* isolates. All the isolates were positive for all the tested genes (100 %; 9/9). These genes are related to IgA protease enzyme production (*zapA*), swarming modulation (*rsbA*), and cytotoxic hemolysin (*hpmA*),[7], [59] as well as *flaA*, which accounts for the production of flagella^26^ and uroepithelial cell adhesion fimbriae (*uca*A),^58^ as well as the *mrpA* gene, which is important for the production of the fimbriae gene,^39^ as shown in Fig. 2.

### 3.4 Sequencing analysis

**Fig. 3:**
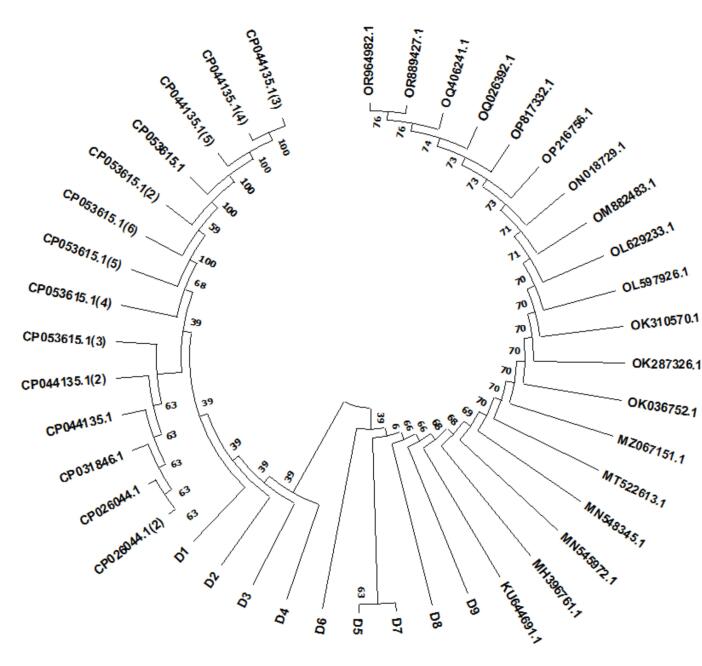
Phylogenetic tree of *16S rRNA* gene sequence alignment of *P. mirabilis* isolates.

The results of alignment via the BLAST tool revealed high similarity of *P. mirabilis* bacteria isolated in this study with the gene bank reference strain. Fig. 3 shows the evolutionary tree that resulted from the alignment of the isolate sequences; furthermore, the neighbour-joining tree method was used to construct the phylogenetic tree. The tree revealed that the nine different isolates (D1--D9) were related to each other and may have been at the same point in evolution at earlier times. The evolutionary history was inferred via the neighbour-joining method. The bootstrap consensus tree inferred from 100 replicates was constructed to represent the evolutionary history of the taxa analyzed. Branches corresponding to partitions reproduced in less than 50 % of the bootstrap replicates are collapsed. The evolutionary distances were computed via the p-distance method and are expressed in units of the number of base differences per site. This analysis involved 42 nucleotide sequences. The codon positions included were 1st + 2nd + 3rd + Noncoding. All positions containing gaps and missing data were eliminated (complete deletion option). There were a total of 654 positions in the final dataset. The *16S rRNA* gene of the strains from Baghdad Province used in this groundbreaking study was compared with those of other species in GenBank (Fig. 3). This tree provides the first theory of the relationships of the study strain with other species. In light of the paucity of molecular data from Iraq, our findings show that the strains share 99 % identity with strains from Korea and China. Our study provides evidence for the molecular genetics of *P. mirabilis* via *16S rRNA* analysis and shows how genomic data can enhance conservation management at the global and local scales. Our results highlight the need for further DNA studies to establish systematic and phylogenetic links between species in Iraq and Asia. Evolutionary analyses were conducted in MEGA11.

## 4 Discussion

The main phenotypic characteristic of *P. mirabilis* is the swarming and smelling of ammonia on XLD agar media according to ISO 6579-1:2017(E), which is different from Salmonella bacteria, which are characterized by a clear colony with a black center and produce H_2_S on XLD agar media. It is possible to find the *Proteus* habitat everywhere; it is an opportunistic human pathogen found in the skin, oral mucosa, gastrointestinal tract, feces, soil, water, and plants.^44^ During the selection of the nominated colony as *Salmonella* spp., another colony is morphologically found and is expected to be *Proteus mirabilis*. *P. mirabilis* is known for its ability to actively obtain antibiotic resistance and infectious toxin genes from other microbial genomes through horizontal gene transfer,^55^ and the high antibiotic resistance rate of *P. mirabilis* may be acquired from *Salmonella* spp. isolated previously.^11^ *Proteus* bacteria are known to be opportunistic human pathogens that are isolated from urine, wounds, and other clinical sources[7], [37]. However, these microorganisms are also frequently implicated in food-borne infections reported worldwide.^25^. Poultry products are among the most important sources of protein for humans, especially in developing countries. Fifty percent of these antibiotics are utilized in livestock globally, leading to the emergence of antibiotic-resistant bacteria[2], [58]. Consequently, the lack of quality assurance, hazard analysis critical control point (HACCP) protocols, and food handling guidelines must be rectified and implemented in Iraqi slaughterhouses throughout the poultry processing chain to mitigate the occurrence of foodborne illnesses as effectively as possible.

A total of 30 % (9/30) of the isolates were *P. mirabilis*, whereas in the study of Sanches et al.^50^^,^ 32 *P. mirabilis* isolates were isolated from 42 chicken carcasses. Compared with the findings of Prasastha Ram et al.^42^, 507 samples were collected from different resources. The results revealed that approximately 34.51 % (175/507) of the *P. mirabilis* isolates were isolated from animal food sources, and a high percentage of *P. mirabilis* isolates were recovered from chicken samples (38.7 %). This variation occurred because several factors, such as the type of sample, antibiotic usage and geographic distribution, play a large role. Furthermore, a study conducted by Sadeq et al.^49^ in Iraq investigated the prevalence of *P. mirabilis* in imported meat from the local markets of Iraqi beef hamburgers, and the results indicate that several types of bacteria exist in beef hamburgers at different percentages, with 13 % *P. mirabilis.*

Molecular identification was performed by amplifying the *16S rRNA* gene, which was positive for *P. mirabilis* in all the isolates; in contrast, a recent study by Jaber and Almiyah^26^ revealed that 80 % of the isolates were confirmed as *P. mirabilis*. *16S rRNA* possesses a significant discrimination ability for bacterial isolation and allows for the identification of living and dead cells but not cultivated cells^41^. Meriem et al.^35^ reported that *16S rRNA* gene sequence analysis relies on a reliable technique to identify bacterial isolates recovered from hospitals and environmental samples.

Additionally, a significant antimicrobial resistance rate of 100 % was observed against nalidixic acid and azithromycin, followed by trimethoprim/sulfamethoxazole, chloramphenicol, and gentamicin at 88.8 %. Similarly, a recent study by Abdellatif et al.^3^ revealed that nalidixic acid and azithromycin were effective against *P. mirabilis* at ratios of 98.4 % and 100 %, respectively. Ronanki et al.^46^ reported that gentamycin susceptibility was high (68.91 %), and chloramphenicol susceptibility was high (14.28 %). Furthermore, a recent study in Iraq performed by Abdullah et al.^4^ in Erbil revealed that the inhibition ability of trimethoprim/sulfamethoxazole was 67.37 %. In our study, the efficacy of ceftazidime and cefotaxime was high against *P. mirabilis*, whereas that of *P. mirabilis* isolates was high in another Iraqi study by Passat.^38^ were shown to be less susceptible to ceftazidime and cefotaxime; in contrast, the results of an additional Iraqi study^8^revealed that the activity of cefotaxime against *P. mirabilis* was 90 %. The most effective antibiotic agents against *P. mirabilis* were meropenem and imipenem (100 %), which agreed with the results of Sohail et al.^56^ and Abdellatif et al.^3^, who revealed that imipenem and meropenem were the most effective antibiotics against *P. mirabilis.*

This study revealed a pattern of resistance (100 %) in all *P. mirabilis* isolates, which was considered multidrug resistance. Antibiotic resistance is a result of a number of factors, including the overuse of antibacterial treatments, improper use of antibiotics, patient-related factors, the prescription habits of doctors, veterinary prescriptions, advertising, over-the-counter antibiotic sales, underuse of microbiological testing, globalization, and improper use of antibiotics, such as using them incorrectly for a short period of time, at the wrong dose, or with the wrong potency^40^. The effects of antibiotic resistance include extended sickness and a higher risk of mortality, longer stays in the hospital and infections, and an increase in the number of diseases that spread in the community^21^. Because doctors perform more invasive surgeries on patients and use more antibiotics to save their lives, nosocomial infections are becoming increasingly prevalent^22^. Antibiotic resistance has been linked to abuse and misuse of these treatments, in addition to a lack of new drug research by the pharmaceutical industry as a consequence of diminished economic incentives and difficult regulatory requirements^61^. Furthermore, the use of antimicrobials as a treatment in the veterinary field may promote the growth of resistant bacteria that are dangerous to people, posing a risk to public health from zoonotic infections such as *Proteus*. Various elements, including soil, water, and crops, interact with bioactive compounds and antibiotic residues in dynamic environments called agroecosystems. The presence of contaminants of emerging concern (CECs), such as hormones, pesticides, antimicrobials, antibiotic-resistant bacteria (ARBs), and antibiotic resistance genes (ARGs), in soils, sediments, and agricultural products is becoming more widespread worldwide^53^. Furthermore, the complex equilibrium between pathogenic and beneficial microorganisms may disrupt the intricate balance of these chemicals, leading to selective pressures that promote the development of antimicrobial resistance^13^. Recent research has demonstrated that exposure to pesticides at sublethal concentrations can increase the spread of ARGs among bacteria, facilitating the development of multidrug resistance more easily^53^. Recently, multidrug resistance (MDR) has significantly increased globally, posing a serious threat to public health. Several recent epidemiological studies have highlighted the emergence of MDR bacterial pathogens, defined as those resistant to at least one agent in three or more antimicrobial classes, from various sources^6^. The high levels of human resistance to antibiotic treatments (MDRs) may not be due to drug abuse and inappropriate use but rather may be spread through food consumption (for example, poultry meat), whose feed on farms has been heavily dosed with therapeutic drugs, particularly antibiotics, to improve and promote their quality and stop the growth and transmission of microbes. Bacteria can obtain resistance genes via mobile elements such as plasmids, which confer adaptability to the host bacterium and facilitate the dissemination of these genes throughout other bacterial populations.

In addition, our study revealed an increase in the incidence and variety of certain additional antibiotic resistance genes in *P. mirabilis*. The *mphA* detection rate was 33.3 %, followed by *that of aac* (6′) (44.4 %), then that of *sul1, floR* and *gyrA* at ratios of 88.8 %, 88.8 % and 100 %, respectively, whereas a recent study by Lv et al.^29^, who isolated *P. mirabilis* from foxes, raccoons and minks in China, reported that the most prevalent resistance genes were *sul1* (94.34 %) and *floR* (88.68 %). The study of the virulence traits of potentially harmful bacteria in animals and animal-derived foods is important for ensuring consumer safety^42^. Furthermore, the current study focused on the identification of virulence genes. Several virulence factors contribute to the pathogenicity of *Proteus* spp.^51^. Gene *zapA* is responsible for 100 % of protein production, which is consistent with the results of Jaber and Almiyah^26^ and Pathirana et al.^39^, who reported that the detection of *zapA* accounted for half of this percentage (50 %). The rotease is considered one of the most essential enzymes because it may degrade IgA and IgG antibodies, lowering the immune response and making bacteria more harmful^15^. The prevalence rate of virulence genes (*rsbA*) and (*mrpA*) in the study of Pathirana et al.^40^ was 45.8 %, whereas our results revealed that the prevalence of these genes was 100 %. Moreover, the hemolysin gene (*hpmA*) was found in 32 (100 %) isolates, and the fimbriae gene (*ucaA*) was found in 16 (50 %) Sanches et al.^50^^,^ which led to crystalline biofilms making bacteria more resistant to antibiotic treatment^29^^,^ whereas this study revealed that the detection of *hmpA* and *uraA* in all the isolates was 100 %. In addition, the flagellar gene *flaA* was detected in all the isolates, which agreed with the findings of Jaber and Almiyah^26^. The presence of *flaA* can carry out swarming motility, which is called the “bull-eye” and is important because it is coupled to the expression of virulence-associated genes (VAGs) and enhances the ability to invade cells^29^^,^ however, Ram et al.^42^ reported the percentages of *flaA* (28.5 %), *hpmA* (60.5 %) and *zapA* (50.28 %) in *P. mirabilis* isolates. Another I study by Ali and Yousif.^9^^,^ who isolated *P. mirabilis* from urinary tract infections in Iraq, reported that *hpmA*, *zapA*, and *flaA* were found at rates of t (%100), (%100) and (%86.66), respectively. This detection may reveal the high virulence ratio that causes illnesses through cross contamination during the processing, chopping and cooking of poultry meat without applying hygiene practices.

All the chicken meat samples collected during this study were contaminated with the same species, *Proteus mirabilis*. The frozen chicken meat samples were collected from the various regions listed in Table 4. According to the phylogenetic tree depicted in Fig. 3, these strains differ from one another; however, they exhibit identical relationships among the isolates. The *P. mirabilis* isolates are interconnected, originating from a common ancestor, and diverging over time. Notably, the D4 isolate shares a node with the D1-D3 isolate, whereas D5 and D7 also evolve over time, likely because of the environmental changes and stressors encountered.

**Table 4:** Sources of the *P. mirabilis* isolated in this study and the region from which *P. mirabilis* was collected.

**Isolate No.**	**Source and region of isolates**
D1	Whole raw frozen 9- piece chicken/Al-Diwaniya
D2	Whole raw frozen 9- piece chicken/Karbala’
D3	Whole raw frozen 9- piece chicken/Sulaymaniyah
D4	Raw frozen chicken breasts/Al-Diwaniya
D5	
D6	Raw frozen chicken breasts/Babel
D7	Raw frozen chicken thighs/Baghdad
D8	Raw frozen chicken thighs/Al-Diwaniya
D9	Raw frozen chicken thighs/Baghdad

Several host and external variables impact the microbiome in chickens, including host species, age, the gut compartment, nutrition, and environmental microbial exposure. The intestinal microbiota of chickens has evolved to include many different microbes from the environment, animals, and humans with whom they contact. This means that the phylogenetic composition of the avian gut microbiota largely overlaps with that of the gut microbiota of humans and other farm animals (^69^).

*Proteus mirabilis* inhabits the digestive tracts of poultry and swine and may contaminate retail meat products through fecal matter during slaughter; additionally, it is widely disseminated in the natural environment and within the intestines of humans and animals. The pathogen *P. mirabilis* is a prevalent opportunistic bacteria responsible for nosocomial infections, especially urinary tract infections[52], [30]). Poultry meat is susceptible to many germs that can cause spoilage during refrigeration, and human illness may arise from handling raw meat, inadequate cooking, or improper management of cooked meats. The presence of *P. mirabilis* in raw meat products has emerged as a significant concern for food safety and human health^30^. Food poisoning incidents attributed to *P. mirabilis* have been documented in China and various other nations^23^. Pathogen cross-contamination of carcasses can transpire at nearly every phase of the process^34^; consequently, to mitigate this significant issue, slaughterhouses should apply a hazard analysis critical control point (HACCP) framework through good manufacturing practices, good hygiene practices and quality control for slaughtering processing and the production of chicken meat, which is increasingly embraced within the poultry sector due to the imperative for a systematic and universally applicable method for food safety management, while quantitative risk assessment (QRA) is employed to address microbiological hazards^30^.

We propose that chicken meat may become contaminated with *P. mirabilis* during carcass evisceration on the slaughter line or by cross-contamination. These isolates are anticipated to be transmitted to consumers, colonize the human gastrointestinal tract, and potentially induce extraintestinal infections, such as urinary tract infections, akin to the presumed effects of *E. coli* originating from food sources.[48], [33], [32], [31] Nonetheless, these bacterial pollutants may endure storage subsequent to carcass processing. Consequently, customers should be aware that chicken flesh may serve as a vector for microbial transmission. Consequently, to reduce cross-contamination, it is imperative to disinfect handling tools after meat processing.^20^

## 5 Conclusion

The current study revealed that domestic frozen chicken carcasses are contaminated with multidrug-resistant *P. mirabilis* strains with high virulence pathogenicity, which poses a challenging threat to public health and is associated with a high risk of food poisoning. Public health stakeholders should monitor and implement a strict food safety management system to limit the misuse of antibiotics on poultry farms and implement the right HACCP system in slaughterhouses to safeguard the public from the spread of multidrug-resistant bacteria to consumers through chicken meat consumption.

## Ethical approval

Not needed.

## CRediT authorship contribution statement

**Zaid A. Thabit:** Methodology, Investigation, Funding acquisition, Formal analysis, Data curation, Conceptualization. **Zahraa A. AlShaheeb:** Writing – original draft, Validation, Software, Resources, Project administration, Methodology, Funding acquisition, Data curation, Conceptualization. **May Ridha Jaafar:** Visualization, Software, Project administration, Methodology, Funding acquisition, Data curation. **Safaa A.S. Al-Qaysi:** Writing – original draft, Visualization, Validation, Funding acquisition. **Sana M.H. Al-Shimmary:** Writing – review & editing.

## Funding

There is no funding.

## Data availability

All bacterial strains were submitted to the National Center for Biotechnology Information (NCBI), and the deposited data are shown within the following links

https://www.ncbi.nlm.nih.gov/nuccore/PQ627873.

https://www.ncbi.nlm.nih.gov/nuccore/PQ627856.

https://www.ncbi.nlm.nih.gov/nuccore/PQ626782.

https://www.ncbi.nlm.nih.gov/nuccore/PQ626779.

https://www.ncbi.nlm.nih.gov/nuccore/PQ626773.

https://www.ncbi.nlm.nih.gov/nuccore/PQ626735.

https://www.ncbi.nlm.nih.gov/nuccore/PQ626724.

https://www.ncbi.nlm.nih.gov/nuccore/PQ626719.

https://www.ncbi.nlm.nih.gov/nuccore/PQ626714.
